# Molecular Characterization of AZD7442 (Tixagevimab-Cilgavimab) Neutralization of SARS-CoV-2 Omicron Subvariants

**DOI:** 10.1128/spectrum.00333-23

**Published:** 2023-03-06

**Authors:** Tiffany L. Roe, Tyler Brady, Nicolette Schuko, Amy Nguyen, Jagadish Beloor, Johnathan D. Guest, Anastasia A. Aksyuk, Kevin M. Tuffy, Tianhui Zhang, Katie Streicher, Elizabeth J. Kelly, Gustavo H. Kijak

**Affiliations:** a Translational Medicine, Vaccines and Immune Therapies, BioPharmaceuticals R&D, AstraZeneca, Gaithersburg, Maryland, USA; b Virology and Vaccine Discovery, Vaccines and Immune Therapies, BioPharmaceuticals R&D, AstraZeneca, Gaithersburg, Maryland, USA; c Data Sciences and Quantitative Biology, BioPharmaceuticals R&D, AstraZeneca, Gaithersburg, Maryland, USA; Fred Hutchinson Cancer Research Center

**Keywords:** AZD7442, tixagevimab, cilgavimab, COVID-19, SARS-CoV-2, monoclonal antibody, Omicron subvariants, mechanism of action

## Abstract

Therapeutic anti-severe acute respiratory syndrome coronavirus 2 (SARS-CoV-2) monoclonal antibodies (MAbs) provide immunosuppressed and vulnerable populations with prophylactic and treatment interventions against coronavirus disease 2019 (COVID-19). AZD7442 (tixagevimab-cilgavimab) is a combination of extended-half-life neutralizing MAbs that bind to distinct epitopes on the receptor binding domain (RBD) of the SARS-CoV-2 spike protein. The Omicron variant of concern carries mutations at >35 positions in the spike protein and has undergone further genetic diversification since its emergence in November 2021. Here, we characterize the *in vitro* neutralization activity of AZD7442 toward major viral subvariants circulating worldwide during the first 9 months of the Omicron wave. BA.2 and its derived subvariants showed the highest susceptibility to AZD7442, while BA.1 and BA.1.1 showed a lower susceptibility. BA.4/BA.5 had a susceptibility level intermediate between BA.1 and BA.2. Mutagenesis of parental Omicron subvariant spike proteins was performed to establish a molecular model to describe the underlying determinants of neutralization by AZD7442 and its component MAbs. The concurrent mutation of residues at positions 446 and 493, located in the tixagevimab and cilgavimab binding sites, was sufficient to enhance *in vitro* susceptibility of BA.1 to AZD7442 and its component MAbs to levels similar to the Wuhan-Hu-1+D614G virus. AZD7442 maintained neutralization activity against all Omicron subvariants tested up to and including BA.5. The evolving nature of the SARS-CoV-2 pandemic warrants continuing real-time molecular surveillance and assessment of *in vitro* activity of MAbs used in prophylaxis against and the treatment of COVID-19.

**IMPORTANCE** MAbs are key therapeutic options for COVID-19 prophylaxis and treatment in immunosuppressed and vulnerable populations. Due to the emergence of SARS-CoV-2 variants, including Omicron, it is vital to ensure that neutralization is maintained for MAb-based interventions. We studied the *in vitro* neutralization of AZD7442 (tixagevimab-cilgavimab), a cocktail of two long-acting MAbs targeting the SARS-CoV-2 spike protein, toward Omicron subvariants circulating from November 2021 to July 2022. AZD7442 neutralized major Omicron subvariants up to and including BA.5. The mechanism of action responsible for the lower *in vitro* susceptibility of BA.1 to AZD7442 was investigated using *in vitro* mutagenesis and molecular modeling. A combination of mutations at two spike protein positions, namely, 446 and 493, was sufficient to enhance BA.1 susceptibility to AZD7442 to levels similar to the Wuhan-Hu-1+D614G ancestral virus. The evolving nature of the SARS-CoV-2 pandemic warrants continuing real-time global molecular surveillance and mechanistic studies of therapeutic MAbs for COVID-19.

## INTRODUCTION

A hallmark of the coronavirus disease-19 (COVID-19) pandemic is the emergence of antigenically distinct variants carrying mutations in the viral spike glycoprotein of severe acute respiratory syndrome coronavirus 2 (SARS-CoV-2), which can impact transmissibility, disease severity, mortality, diagnostic detection, and effectiveness of vaccines and therapeutic monoclonal antibodies (MAbs) (1). Globally, vaccines have been widely effective for reducing the disease burden of COVID-19 (2–6). However, there remains a substantial population of immunocompromised individuals who do not elicit an adequate immune response to vaccination and so remain at risk of severe disease (7). Prophylaxis and treatment with anti–SARS-CoV-2 MAbs offer immunosuppressed and vulnerable populations with additional interventions against COVID-19 (8, 9).

AZD7442 is a combination of two fully human SARS-CoV-2-neutralizing MAbs, namely, tixagevimab (AZD8895) and cilgavimab (AZD1061), derived from potent antibodies isolated from B cells of individuals with prior SARS-CoV-2 infection (10). Tixagevimab and cilgavimab carry a triple modification (L243F/L235E/P331S) that reduces fragment crystallizable effector function and the risk of antibody-dependent disease enhancement and carry a YTE modification (M257Y/S259T/T261E) that extends the half-life of each MAb (11). Following administration, the AZD7442 component MAbs bind to distinct epitopes on the receptor-binding domain (RBD) of the SARS-CoV-2 spike protein, preventing viral interaction with the cellular receptor human angiotensin-converting enzyme 2 (ACE2) and potently neutralizing the virus. The effectiveness of AZD7442 for prophylactic and treatment usage has been demonstrated in phase 3 clinical trials (9, 12). The AZD7442 cocktail has retained *in vitro* susceptibility to historical variants of concern (VOCs) and variants of interest (13, 14).

The Omicron VOC first emerged in November 2021, and by January 2022, it had spread rapidly worldwide, replacing the Delta variant, which had dominated the pandemic during the previous 6 months (15). As evidenced by real-time molecular surveillance, Omicron has undergone genetic diversification into subvariants with marked differences in the spike protein that impact both structure and function, including a modulation of the affinity to ACE2 (16–19). These changes are reflected in distinct *in vitro* reactivity profiles toward therapeutic MAbs (20–26). Here, we characterize the *in vitro* neutralization potency of AZD7442 toward major subvariants circulating worldwide from November 2021 to July 2022 and provide a molecular model to describe the underlying mechanism of action.

## RESULTS

### SARS-CoV-2 spike protein diversity.

**FIG 1:**
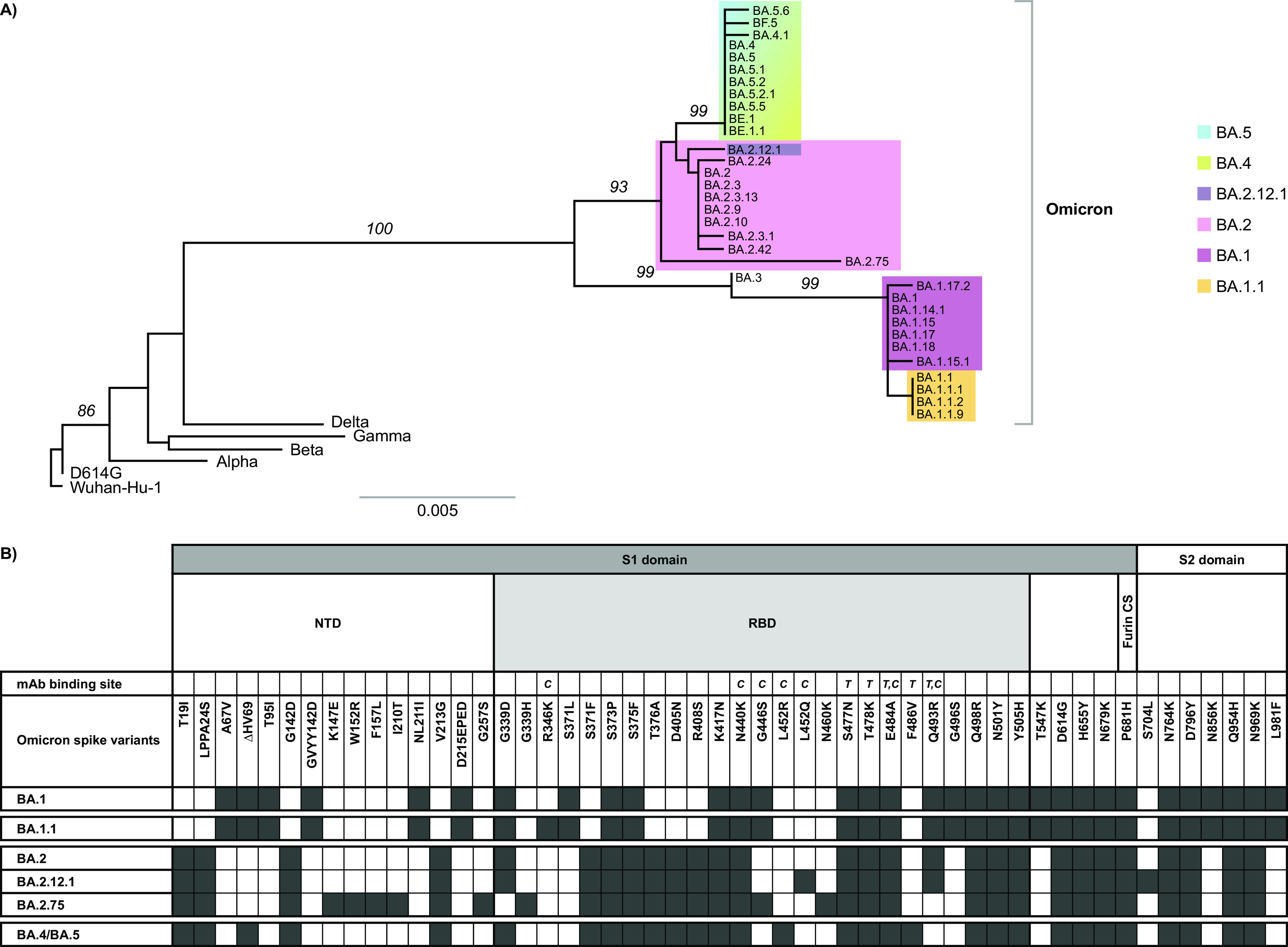
SARS-CoV-2 spike protein diversity. (A) Phylogenetic tree of representative subvariants circulating during the first 9 months of the Omicron wave. Major Omicron subclusters BA.1/BA.1.1, BA.2, and BA.4/BA.5 are highlighted. Pre-Omicron variants of concern, and reference sequences Wuhan-Hu-1 and Wuhan-Hu-1+D614G (D614G), are shown to evidence the divergence of Omicron subvariants from ancestral strains. The horizontal scale represents 0.5% *p*-distance. Bootstrap-support values of ≥70% are shown above branches. (B) Omicron subvariant-defining signatures in the SARS-CoV-2 spike protein. Location of the spike N-terminal domain (NTD), receptor-binding domain (RBD), and furin cleavage site (CS), as well as the binding sites of AZD7442 component MAbs tixagevimab (T) and cilgavimab (C) are shown for reference. While both tixagevimab and cilgavimab bind at spike positions 484 and 493, each MAb makes contact with different atoms in the residues, allowing for cooperative binding (10).

Omicron sequences are phylogenetically distinct from previously circulating SARS-CoV-2 lineages and are characterized by a larger number of mutations from the ancestral Wuhan-Hu-1 virus, especially in the gene encoding the spike glycoprotein (Fig. 1). In addition to numerous mutations in the spike N-terminal domain and S2 domain, Omicron sequences present with 15 to 17 amino acid substitutions in the RBD, a major target of neutralizing antibodies.

Ongoing Omicron circulation has led to its diversification into distinct subvariants, which can be divided into the following three major subclusters based on analysis of the spike protein: BA.1/BA.1.1, BA.2, and BA.4/BA.5. Spike sequences of subvariants BA.1 (the original B.1.1.529 clade) and BA.1.1 are highly similar, differing in only one position. However, the BA.2 subvariant presents major differences from BA.1 throughout the spike, and its RBD differs from BA.1 in six positions. Within the BA.2 subcluster, the RBDs of BA.2.12.1 and BA.2.75 differ from the BA.2 subvariant at one and four positions, respectively. BA.4 and BA.5 subvariants share identical spike sequences and are closer to the BA.2 subcluster than to BA.1/BA.1.1. In addition, there are subvariants that have circulated at low levels in which spike protein sequences resemble chimeras between BA.1 and BA.2 (e.g., BA.3).

**FIG 2:**
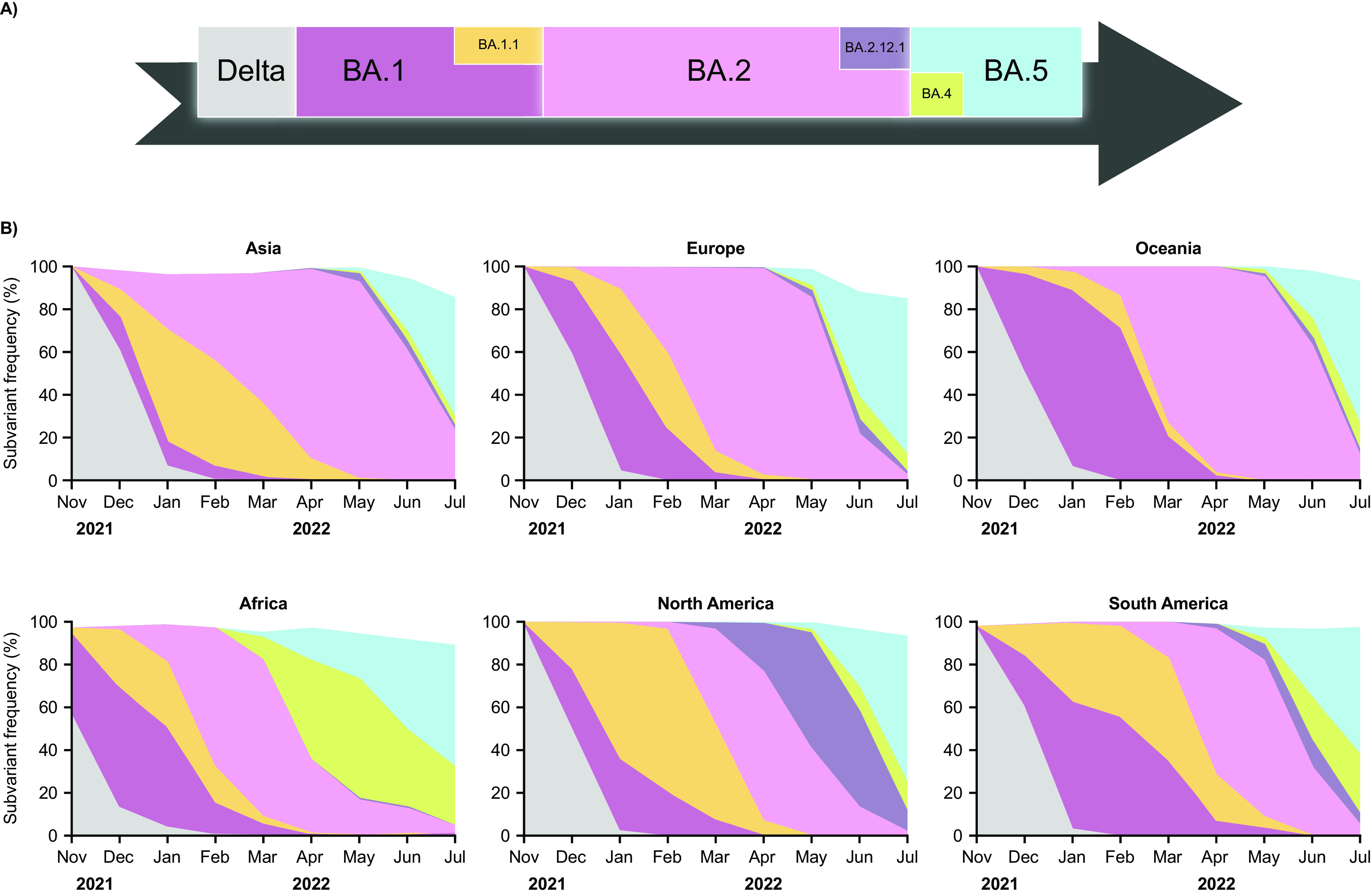
Temporal dynamics of dominant Omicron subvariants, stratified by region. The arrow represents the global profile and the subvariant color coding. Pango lineages were grouped within their respective parental subvariants BA.1, BA.1.1, BA.2, BA.2.12.1, BA.4, and BA.5 (see Materials and Methods for details). The pre-Omicron variant of concern Delta is shown for reference.

Real-time global SARS-CoV-2 molecular surveillance has revealed a dynamic temporal profile defined by the succession of emerging subvariants that replace previously dominant variants, as follows: BA.1/BA.1.1→BA.2→BA.5 (Fig. 2). Overall, this theme has been reiterated worldwide, albeit with slight regional differences. For instance, between the phases of dominance of BA.2 and BA.5, Africa and North America experienced high-level circulation of BA.4 and BA.2.12.1, respectively, while these two subvariants circulated elsewhere at lower levels or for shorter periods.

### *In vitro* susceptibility of Omicron subvariants to AZD7442.

**FIG 3:**
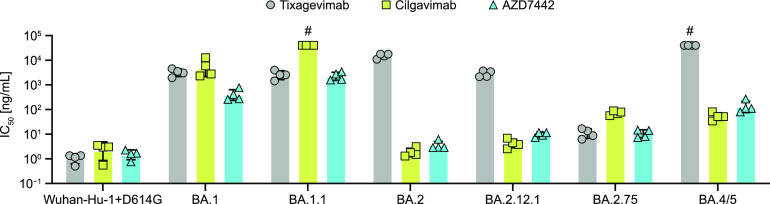
*In vitro* susceptibility of Omicron subvariants to AZD7442 and its component MAbs (i.e., tixagevimab and cilgavimab). Bars represent the geometric mean of IC_50_ values obtained in the microneutralization assay using SARS-CoV-2 spike pseudotyped lentiviral particles. *#*, IC_50_ values greater than the upper limit of quantitation.

Core Omicron mutations (i.e., N440K, S477N, T478K, and E484A) and subvariant-specific mutations (i.e., R346K, G446S, and Q493R) occur within the binding sites of tixagevimab and cilgavimab. To define the impact of Omicron genetic diversity on the *in vitro* potency of AZD7442 and its MAb components, the susceptibility of major Omicron subvariants was tested in a spike-pseudotyped microneutralization assay. Subvariant BA.1 showed a reduction in susceptibility to AZD7442 (50% inhibitory concentration [IC_50_], 389.2 ng/mL) compared with the Wuhan-Hu-1+D614G reference (IC_50_, 1.5 ng/mL) (Fig. 3; see Table S1 in the supplemental material). Notably, the AZD7442 cocktail showed higher potency toward these BA.1 subvariants compared with each of the individual MAb components separately. Subvariant BA.1.1, carrying an additional mutation, R346K, in the cilgavimab binding site, showed a measurable but lower susceptibility to AZD7442 (IC_50_, 2249.1 ng/mL). A marked increase in AZD7442 potency was observed toward subvariants of the BA.2 cluster (IC_50_, 3.5 to 10.6 ng/mL), at levels similar to the Wuhan-Hu-1+D614G reference. For subvariants BA.2 and BA.2.12.1, the increase in AZD7442 susceptibility was driven by cilgavimab (IC_50_, 1.8 to 4.2 ng/mL). Among Omicron subvariants, BA.2.75 showed the highest susceptibility to tixagevimab (IC_50_, 10.6 ng/mL). In the case of BA.4/BA.5, which carries mutation F486V in the tixagevimab binding site, susceptibility to AZD7442 (IC_50_, 128.9 ng/mL) was mediated by cilgavimab (IC_50_, 52.3 ng/mL). All Omicron subvariants tested up to and including BA.5 were neutralized by AZD7442.

### Key RBD residues mediating AZD7442 neutralization to Omicron subvariants.

**FIG 4:**
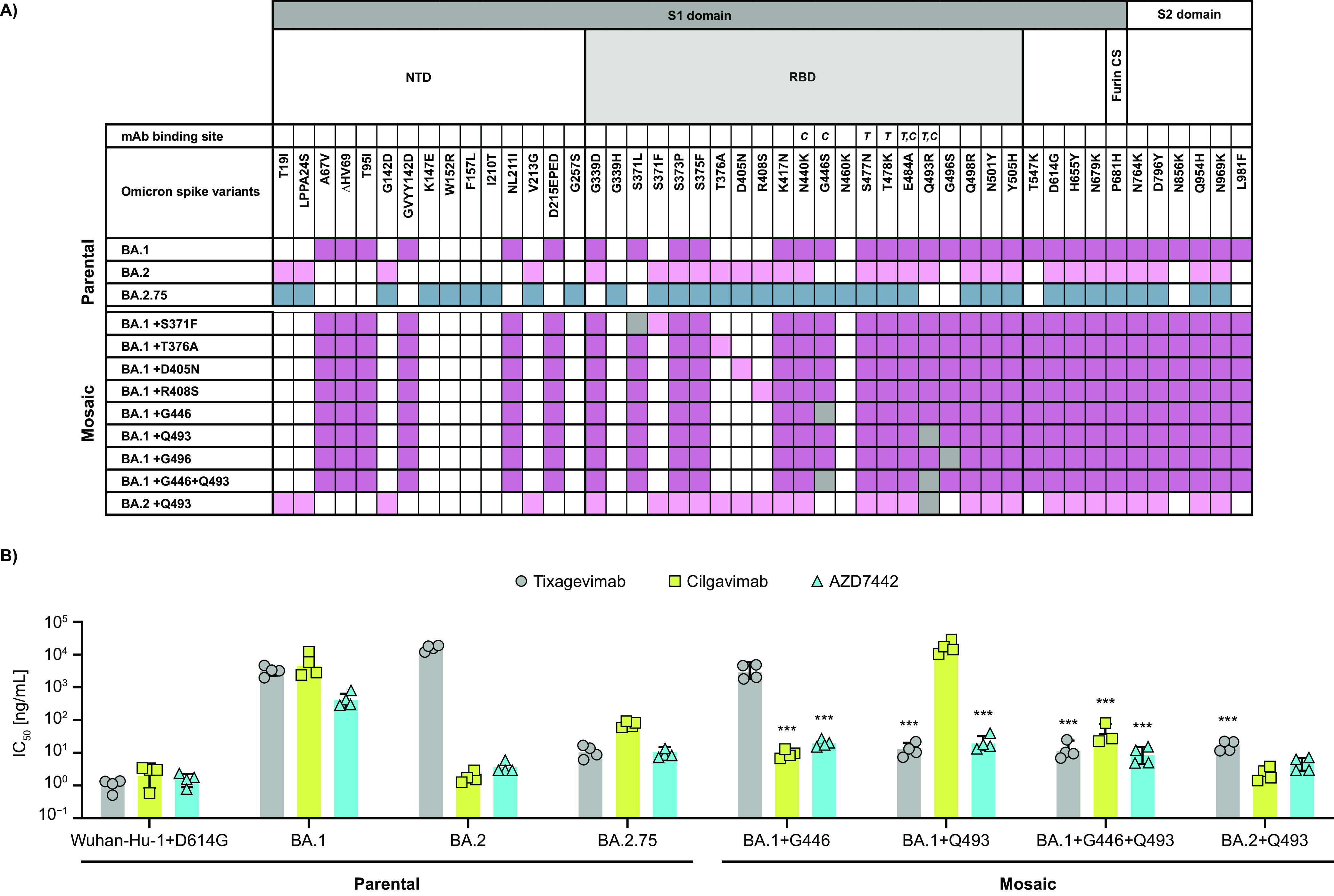
Impact of mutagenesis of key residues in Omicron receptor binding domain (RBD) on *in vitro* susceptibility to AZD7442 and its component MAbs (i.e., tixagevimab and cilgavimab). (A) SARS-CoV-2 spike protein mosaic constructs incorporating BA.2 (pink) and BA.2.75 (blue)-defining signatures in the background of BA.1 (purple) and BA.2 subvariants. Mutations resulting in a reversion to the Wuhan-Hu-1 sequence are highlighted in gray. Spike protein domains and the location of binding sites of tixagevimab (T) and cilgavimab (C) are indicated. (B) IC_50_ values of BA.1 and BA.2 parental and mosaic mutants obtained in the microneutralization assay using SARS-CoV-2 spike pseudotyped lentiviral particles. Only mosaic mutants with the highest impact of AZD7442 susceptibility are shown (see Fig. S1 for other assessed mutants). ***, *P* < 0.001; NTD, N-terminal domain; CS, cleavage site.

To investigate the potential mechanism mediating distinct neutralization profiles of tixagevimab, cilgavimab, and AZD7442 to BA.1, BA.2, and BA.2.75, the impact of subvariant-specific signatures in the RBD was assessed. First, determinants of BA.1 *in vitro* susceptibility to cilgavimab and AZD7442 were studied. In the context of the BA.1 spike, individual constructs separately carrying individual BA.2-signature RBD mutations (i.e., S371F, T376A, D405N, R408S, G446, and G496) were tested for *in vitro* neutralization by AZD7442 and its MAb components (Fig. 4A). Of note, BA.2 signatures G446 and G496 represent reversions of BA.1 mutations G446S and G496S. Out of the six evaluated mutations, the introduction of mutation G446 in the background of BA.1 rendered the greatest increase in susceptibility to AZD7442 (19.5-fold change compared with unmodified BA.1), mediated by an increase in cilgavimab potency (524.8-fold change), but with no change in susceptibility to tixagevimab (Fig. 4B; see Table S2 in the supplemental material). An increase in susceptibility to AZD7442 was also observed when mutations D405N, R408S, and G496 were introduced individually in the background of BA.1 (see Fig. S1 in the supplemental material; Table S2), but they were of lower magnitude (2.9-, 1.8-, and 2.7-fold change versus BA.1, respectively).

The molecular basis for distinct susceptibility to tixagevimab observed for BA.1 and BA.2 versus BA.2.75 was investigated. A major difference between the spike protein sequences of these subvariants occurs at position 493 in the tixagevimab binding site, with BA.2.75 encoding glutamine and BA.1/BA.2 encoding arginine. While both tixagevimab and cilgavimab bind at position 493, each MAb makes contact with different atoms in the residue, allowing for cooperative binding. Of note, BA.2.75 signature Q493 represents a reversion of BA.1/BA.2 mutation Q493R. The introduction of Q493 resulted in a 248.2-fold increase in susceptibility to tixagevimab compared with unmodified BA.1, which led to a 19.3-fold increase in susceptibility to AZD7442 but no change in susceptibility to cilgavimab. The introduction of the same mutation in BA.2 resulted in a 943.5-fold increase in susceptibility to tixagevimab compared with unmodified BA.2, with the susceptibility to cilgavimab and AZD7442 remaining at levels similar to Wuhan-Hu-1+D614G.

Overall, these results indicate that the separate introduction of mutations G446 and Q493 confer an increase in BA.1 susceptibility to cilgavimab and tixagevimab, respectively, with no impact on susceptibility to the other MAbs. Parental BA.2 encodes G446 (Fig. 1), and so the susceptibility of the BA.2+Q493 mutant to tixagevimab, cilgavimab, and AZD7442 supports the functional compatibility of G446 and Q493 mutations. To explore if this effect could be extended to BA.1, a G446+Q493 mutant was generated in the background of BA.1. Compared with parental BA.1, BA.1+G446+Q493 showed 272.4-, 136.8-, and 47.7-fold increases in *in vitro* susceptibility to tixagevimab, cilgavimab, and AZD7442, respectively, confirming the key role of these residues in the contexts of BA.1 and BA.2.

Of note, mutation G446S is a signature of BA.1/BA.1.1, BA.2.75, and their derived subvariants but is not present in other major BA.2-derived subvariants or in BA.4/BA.5 (Fig. 1). Prior to the emergence of the Omicron variant, mutation G446S was present in <0.02% of sequences, and once the incidence of BA.1/BA.1.1 decreased, so did the incidence of mutation G446S (see Fig. S2A in the supplemental material). Similarly, mutation Q493R is a signature of BA.1/BA.1.1 and most BA.2-derived subvariants, excluding BA.2.75. Prior to the emergence of the Omicron variant, mutation Q493R was present in <0.02% of sequences, and once the incidence of BA.1/BA.1.1 and BA.2 decreased, so did the incidence of mutation Q493R (Fig. S2B).

### Structural analysis of the impact of variation at SARS-CoV-2 spike residues 446 and 493 on AZD7442 neutralization.

**FIG 5:**
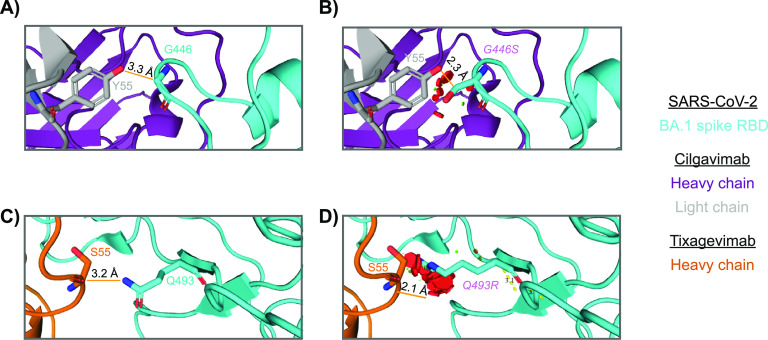
*In silico* structural modeling of the impact of mutations at SARS-CoV-2 spike positions 446 and 493 on AZD7442 binding. The G446S mutation clashes with the cilgavimab light chain. (A) The G446 residue in the SARS-CoV-2 RBD (cyan) is shown contacting light chain CDRL2 (gray) residue Y55 in cilgavimab. The cilgavimab heavy chain is shown in purple. (B) Modeled mutation G446S (magenta) in antibody-bound backbone shows a probable clash between the 446 side chain and Y55, as indicated by the red and green circles output by PyMOL. The gold dashed line shows the shortest distance between an atom on Y55 and an atom on G446 or modeled G446S. Mutation Q493R clashes with the tixagevimab heavy chain. (C) The Q493 residue in SARS-CoV-2 RBD (cyan) is shown contacting heavy chain CDRH2 residue Y55 in tixagevimab (orange). (D) Modeled mutation Q493R (magenta) in antibody-bound backbone shows a probable clash between 493 side chain and S55, as indicated by the red, green, and yellow circles. The gold dashed line shows the shortest distance between an atom on S55 and an atom on Q493 or modeled Q493R. Modeling based on PDB 7L7E (10). All visualizations, distance calculations, and mutagenesis were conducted in PyMOL. See Materials and Methods for details.

To examine the structural basis of observed *in vitro* susceptibility shifts associated with mutations at spike positions 446 and 493, *in silico* mutagenesis analyses were conducted. First, the glycine at residue 446 in a published structure of RBD in complex with tixagevimab and cilgavimab (10) was mutated to a serine to analyze the potential structural impact of this BA.1-signature mutation on antibody binding (Fig. 5A and B). In a structure with an RBD from Wuhan-Hu-1, G446 forms a close contact with a tyrosine (i.e., Y55) in a light chain complementarity determining region (CDR) of cilgavimab. The side chain of Y55 is within 4 Å of the G446 backbone. Substituting the glycine side chain by a larger serine side chain (i.e., G446S) affects the distance to Y55, which was reduced to 2.3 Å, leading to predicted clashes between the side chains of the serine in the RBD and tyrosine in cilgavimab.

A similar approach was used to evaluate the interaction of residue Q493, mutated to Q493R in the AZD7442-bound RBD structure (Fig. 5C and D). As noted previously, residue 493 contacts both tixagevimab and cilgavimab, with Q493 backbone atoms contacting the cilgavimab light chain and Q493 side chain atoms contacting the tixagevimab heavy chain. A serine in a heavy chain CDR of tixagevimab (i.e., S55) is within 4 Å of the Q493 side chain. When Q493R from BA.1 and BA.2 was introduced, the larger side chain of arginine was only 2.1 Å from the S55 side chain, leading to multiple predicted clashes in this interaction, impacting binding. As expected, the backbone interactions with cilgavimab are less sensitive to side chain differences, consistent with no impact on cilgavimab susceptibility in the presence of mutation Q493R.

In addition, mutations to spike residues 405, 408, and 496 were examined according to their effects on antibody susceptibility *in vitro*. As these residues do not occur within the AZD7442 binding sites, their direct structural effects on antibody susceptibility could not be discerned in this analysis (data not shown).

## DISCUSSION

SARS-CoV-2 genetic diversification has led to the emergence of novel viral variants, of which some have been declared as VOCs by the World Health Organization (WHO) due to their global public health significance (27). Notably, the first 9 months of the Omicron wave was a period of high incidence of SARS-CoV-2 infections, with 57% of the cases that occurred since the beginning of the pandemic being accrued between November 2021 and July 2022 (28). Due to the antigenic diversity represented by Omicron and the distinct subvariants into which it further evolved, the effectiveness of MAb-based therapies against these variants has been a major area of investigation. The current *in vitro* susceptibility assessment shows that AZD7442 neutralizes all Omicron subvariants tested up to and including BA.5, in concordance with previous reports (21, 29–35).

Subvariant-specific signatures in the RBD of the Omicron spike include positions in the binding sites of tixagevimab and cilgavimab (10), which result in distinct profiles of *in vitro* susceptibility to AZD7442 and its component MAbs. BA.2 and its derived subvariants showed the highest susceptibility to AZD7442, with IC_50_ values at the level of the Wuhan-Hu-1+D614G reference strain, while BA.1 and BA.1.1 showed lower susceptibility. BA.4/BA.5 had a susceptibility level intermediate between BA.1 and BA.2. Interestingly, BA.2 and BA.2.75 have comparable susceptibilities to AZD7442, but BA.2 is more susceptible to cilgavimab, whereas BA.2.75 is more susceptible to tixagevimab. These results reiterate the pattern by which AZD7442 potency is maintained across variants, with a decrease in susceptibility to one of the MAb components usually being compensated by an increase in susceptibility to the second MAb in the cocktail.

Compared with the ancestral Wuhan-Hu-1 strain, the spike protein of subvariant BA.1 carries mutations at >35 positions, which likely emerged by and are being maintained through epistatic networks (36, 37). A detailed mechanism of action analysis based on introduction of subvariant-specific signatures into spikes of parental Omicron subvariants showed that concurrent mutations at positions 446 and 493 in the binding sites of tixagevimab and cilgavimab were sufficient to enhance the *in vitro* susceptibility of BA.1 to AZD7442 and its component MAbs to levels similar to the Wuhan-Hu-1+D614G virus. The proposed mechanism of action, supported by *in silico* modeling of the SARS-CoV-2 spike RBD bound to tixagevimab and cilgavimab, is the generation of steric hinderance between the side chains of G446S and Q493R in the viral spike of BA.1 and AZD7442 component MAbs, which is relieved by mutations G446 and Q493, consistent with previous reports (38).

Spike mutations G446S and Q493R first emerged concurrently with the rise of Omicron in November 2021, and their incidence decreased to pre-Omicron levels after BA.1 and BA.1.1 were replaced by BA.2 (April 2022) and after BA.2 and BA.2.12.1 were replaced by BA.5 (July 2022), respectively. Overall, the temporal profiles of these mutations ascribe to the general dynamics of the pandemic, defined by the emergence of variants followed by their replacement by other variants with different antigenic profiles.

Variant substitutions within the MAb binding sites are understandably most well-recognized for their ability to influence changes in neutralizing potency. The current mutagenesis analyses suggest that variation in RBD positions outside the binding sites of tixagevimab and cilgavimab (i.e., D405N, R408S, and G496) can also increase the susceptibility of variants, notably of BA.1 to cilgavimab, albeit to a lesser degree than mutation G446. The impact of these mutations may be mediated by RBD-ACE2 interactions, indirect effects to antibody binding, or modulation of inter-RBD interactions in the context of the SARS-CoV-2 spike (16, 17). For instance, position 496 has been identified as a key residue for the binding of Omicron spike to ACE2, whereby BA.1 affinity to ACE2 is attenuated when G496S is present by modulating the interaction of nearby RBD and ACE2 residues (16). Mutation G496S is present in BA.1 but is absent in BA.2, and a glycine at that position results in an increased fitness *in vitro* and in an animal model compared with serine (39).

The first 9 months of the Omicron wave were characterized by the succession of 4 to 5 phases, with each defined by the dominance of a single subvariant. In contrast, the period of August to November 2022 has been marked by the cocirculation of subvariants with diverse levels of susceptibility to therapeutic MAbs, after the emergence of multiple Omicron subvariants (e.g., BA.2.75.2, BA.5.2.6, BF.7, BF.11, BN.1, BQ.1, BQ.1.1, and XBB) circulating in the milieu of BA.5. Unlike the first phases of the Omicron wave, no single novel subvariant has yet become dominant globally. The potential impact of this new SARS-CoV-2 genetic landscape on viral susceptibility to therapeutic MAbs calls for continued investigation in clinical trials and real-world effectiveness studies.

In conclusion, AZD7442 shows *in vitro* neutralization against major subvariants circulating during the first 9 months of the Omicron wave, up to and including BA.5. The current analysis has defined, in the context of Omicron subvariants, the key roles of spike protein amino acids at positions 446 and 493 in the mechanism of action of AZD7442 component MAbs. The evolving nature of the SARS-CoV-2 pandemic warrants continuing real-time molecular surveillance and assessment of *in vitro* activity of MAbs used in prophylaxis against and in the treatment of COVID-19.

## MATERIALS AND METHODS

### SARS-CoV-2 molecular surveillance.

To define the temporal dynamics of SARS-CoV-2 variants, metadata from viral sequences deposited in the Global Initiative on Sharing Avian Influenza Data (GISAID) database (40) with collection dates between 01 November 2021 and 31 July 2022 (*n *= 5,118,798 sequences) were accessed through the COVIDCG pipeline (41) (https://covidcg.org/, accessed on 27 August 2022). Annotated lineages were grouped based on their parental lineages/Omicron subvariant (i.e., Delta, BA.1, BA.1.1, BA.2, BA.2.12.1, BA.4, and BA.5) following the Pango nomenclature definition (42) (https://cov-lineages.org/, accessed on 27 August 2022). To accommodate for regional differences, monthly subvariant incidences were computed separately for Africa, Asia, Europe, North America, Oceania, and South America.

The temporal dynamics of SARS-CoV-2 spike mutations G446S and Q493R in sequences collected between 15 December 2019 and 31 July 2022 were computed using the “Compare Locations” function in COVIDCG (https://cov-lineages.org/, accessed on 24 November 2022).

### Phylogenetic analysis.

SARS-CoV-2 spike protein consensus sequences of the Alpha, Beta, Gamma, and Delta VOCs and of representative Omicron subvariants (i.e., subvariants that had achieved a monthly incidence of ≥5% in at least one of the global regions in the studied time period) were obtained from COVIDCG (https://cov-lineages.org/, accessed on 18 September 2022) using the “Lineage Report” function. Protein sequences were aligned using Clustal Omega 1.2.3 (43) implemented in Geneious Prime 2022.1.1 (https://www.geneious.com). Phylogenetic relations were inferred using the neighbor-joining method based on p-distances (44) as implemented in MEGA11 (45). The resulting phylogenetic tree was rooted at the Wuhan-Hu-1 reference strain (GenBank accession number NC_045512.2) and was visualized using FigTree (version 1.4.4; distributed by the author online through http://tree.bio.ed.ac.uk/software/figtree/, accessed on 15 August 2022). The reliability of the phylogenetic tree was assessed using bootstrapping (46) implemented in MEGA11 (number of replicates, 1,000).

### Plasmids and cell lines.

Inserts encoding residues 1 to 1,254 of the SARS-CoV-2 spike protein were designed through codon optimization of the consensus sequences of Omicron subvariants under study (see Table S3 in the supplemental material) and were incorporated in the pCAGG-Sdl19 plasmid. Mosaic variants were designed based on the comparison of the protein sequence alignment of RBDs of BA.1, BA.2, and BA.2.75 to survey for mutations of interest (see Table S4 in the supplemental material). Mosaic mutant naming follows a nomenclature based on the amino acid sequence of the Wuhan-Hu-1 reference strain (GenBank accession number NC_045512.2), and so reversions of spike mutations G446S, G496S, and Q493R are denoted as G446, G496, and Q493, respectively. All plasmids were synthesized at GenScript (Piscataway, NJ).

The Freestyle 293FT cells (Invitrogen, Waltham, MA) were cultured at 37°C and 8% CO_2_ in Freestyle 293 expression medium (Gibco, Waltham, MA). The stably transfected HEK-Blue-ACE2/TMPRSS2 (InVivogen, San Diego, CA) cells were cultured at 37°C and 5%CO_2_ in Dulbecco’s modified eagle medium (DMEM) supplemented with 10% fetal bovine serum (FBS), Glutamax, and 1× penicillin-streptomycin (pen-strep) along with required selection antibiotics, namely, zeocin (100 μg/mL), puromycin (0.5 μg/mL), and hygromycin (200 μg/mL), according to the manufacturer’s instructions.

### Antibodies.

Nonclinical preparations of tixagevimab and cilgavimab were synthesized by AstraZeneca Cell Culture and Fermentation Sciences (Gaithersburg, MD) and purified by AstraZeneca Purification Process Sciences (Gaithersburg). Cells expressing either cilgavimab or tixagevimab were generated by stably transfecting an in-house Chines hamster ovary (CHO) cell line with the appropriate expression vector followed by the establishment of a clonal cell line for clinical and nonclinical manufacturing. The antibodies were produced in bioreactors using animal component-free growth medium, nutrient feeds, and supplements. The drug substances were purified using protein A column chromatography and a low pH virus inactivation step. Final antibody concentrations were determined by measuring absorbance at 280 nm. AZD7442 was obtained by combining equimolar amounts of tixagevimab and cilgavimab.

### SARS-CoV-2 pseudovirus production and infectivity.

SARS-CoV-2 spike pseudotyped lentiviral particles were generated using a third-generation HIV-based lentiviral vector system expressing luciferase as reported previously (47), with several modifications. FreeStyle 293FT cells (Invitrogen, Waltham, MA), at a density of 1 × 10^6^ cells/mL in FreeStyle medium, were transfected with plasmids encoding the SARS-CoV-2 spike sequence, the lentiviral vector expressing luciferase pESRC-CMV-Luc2p-EFI, and the packaging plasmids pPACKH1Gag-pol and pAZRev, using 293fectin reagent (Gibco, Carlsbad, CA), per the manufacturer’s instructions.

Supernatants were harvested at 48 h, passed through a 0.45-μm filter, concentrated by ultracentrifugation (25,000 rpm for 2 h) or 10% sucrose cushion/high-speed centrifugation (10,000 × *g* for 4 h), and resuspended in Opti-MEM (Gibco, Waltham, MA). Pseudoviral particle stocks were assayed to quantify median tissue culture infectious dose/mL (TCID_50_/mL) and stored at −80°C until tested for neutralization.

Pseudovirus infectivity was determined by measuring the luminescence of pseudovirus-generated luciferase titrated in HEK-Blue-ACE2/TMPRSS2 cells and expressed as relative luminescence units (RLUs) per viral input volume read on an EnVision 2105 multimode plate reader (PerkinElmer, Akron, OH) using the Bright-Glo luciferase assay system (Promega, Madison, WI), according to the manufacturer’s instructions. TCID_50_/mL was calculated based on the Spearman-Karber method (48), with wells with signals of >5,000 RLUs being considered positive events.

### Pseudovirus microneutralization assay.

*In vitro* susceptibility of lentiviral particles pseudotyped with the SARS-CoV-2 spike protein was assessed as reported previously (47), with several modifications. AZD7442 and its individual components (tixagevimab and cilgavimab) were serially diluted in assay media (DMEM, 10%FBS, and Glutamax) from a starting concentration of 1.5 μg/mL or 40.5 μg/mL in a 384-well black plate. The MAbs were incubated in the presence of pseudovirus at a targeted multiplicity of infection of 0.25 for 0.5 h. Then, HEK-Blue-ACE2/TMPRSS2 cells were plated at a density of 2 × 10^3^ cells/well in the presence of 1 μg/mL of Polybrene. Plates were incubated at 37°C for 48 h. The Bright-Glo luciferase assay system (Promega, Madison, WI) was used according to the manufacturer’s recommendations to develop plates that were then read on an EnVision 2105 multimode plate reader (PerkinElmer, Akron, OH). Percent inhibition was calculated by normalization to infectivity/positive control. The IC_50_ values were determined by nonlinear regression (GraphPad Software, San Diego, CA; version 9.0.0). The average IC_50_ was determined by a minimum of four independent experiments. The SARS-CoV-2 Wuhan-Hu-1/2019+D614G spike pseudovirus was run in each experiment and was used as a reference to calculate the IC_50_-fold change for tixagevimab, cilgavimab, and AZD7442.

### Structural analysis.

A structure of SARS-CoV-2 RBD bound to tixagevimab and cilgavimab (Protein Data Bank [PDB] 7L7E [10]) was downloaded from the Protein Data Bank (49). The structure was visualized in PyMOL version 2.5 (Schrödinger) during analysis. The measurement wizard in PyMOL was used to find and quantify the shortest distance between any atom in residue 446 or 493 and any atom in tixagevimab or cilgavimab. The mutagenesis wizard in PyMOL was used to introduce G446S or Q493R in the existing structure. Red, green, or yellow disks were automatically output by PyMOL when each mutation was introduced, indicating likely clashes with other atoms in the structure due to overlapping van der Waals interactions (https://pymolwiki.org/index.php/Mutagenesis). When Q493R was introduced, the mutagenesis wizard also showed distances of Q493R backbone atoms to a nearby residue in RBD; these data did not factor into the analysis.

### Statistical analysis.

A linear model was fitted to log-transformed IC_50_ values for each MAb separately in R version 4.1.3 (R Core Team, Vienna, Austria). To test the hypothesis that the introduction of mutations in the BA.1 and BA.2 background led to an increase in susceptibility to the tested MAbs, the IC_50_ value of each mosaic mutant for each MAb was compared to the corresponding IC_50_ value of its parental subvariant, via one-sided *t* test. Unadjusted *P* values were calculated.
