# Supplementary material for: Molecular Characterization of AZD7442 (Tixagevimab-Cilgavimab) Neutralization of SARS-CoV-2 Omicron Subvariants
Source: Microbiol Spectr. 2023 Mar 6;11(2):e00333-23. doi: 10.1128/spectrum.00333-23 (PMC10100701; doi:10.1128/spectrum.00333-23)
Supplement: Supplemental file 1 — Supplemental material. Download spectrum.00333-23-s0001.pdf, PDF file, 0.7 MB [file spectrum.00333-23-s0001.pdf]

Supplementary Materials for

Manuscript: "*Molecular Characterization of AZD7442 (Tixagevimab-Cilgavimab) Neutralization of SARS-CoV-2 Omicron Subvariants*"

This file includes:

Supplemental Table S1 to Supplemental Table S4, and Supplemental Figure 1 and Supplemental Figure 2

SUPPLEMENTAL TABLE 1 *In vitro* susceptibility of Omicron subvariants to AZD7442 and its component mAbs (i.e., tixagevimab and cilgavimab). See text for details

SARS-CoV-2 subvariant	Tixagevimab		Cilgavimab		AZD7442	
	IC_{50} (ng/mL)	Fold change[a]	IC_{50} (ng/mL)	Fold change[a]	IC_{50} (ng/mL)	Fold change[a]
Wuhan-Hu-1+D614G	1.0	1.0	2.1	1.0	1.5	1.0
BA.1	3137.5	3055.7	4712.8	2271.9	389.2	262.5
BA.1.1	2496.1	2431.0	>40,500	>19,523	2249.1	1516.6
BA.2	15,150.6	14,755.6	1.8	0.9	3.5	2.4
BA.2.12.1	2786.4	2713.8	4.2	2.0	9.8	6.6
BA.2.75	10.6	10.3	72.7	35.1	10.6	7.1
BA.4/5	>40,500	>39,444	52.3	25.2	128.9	86.9

[a]IC_{50} subvariant/IC_{50} Wuhan-Hu-1+D614G. $IC_{50,}$ 50% inhibitory concentration; mAbs, monoclonal antibodies.

SUPPLEMENTAL TABLE 2 *In vitro* susceptibility of SARS-CoV-2 spike mosaic mutants in the background of BA.1 and BA.2 subvariants to AZD7442 and its component mAbs (i.e., tixagevimab and cilgavimab). See text for details

**Mosaic mutant**	**Tixagevimab**			**Cilgavimab**			**AZD7442**		
	**IC_{50} (ng/mL)**	**Fold change[a]**	***p*-value[b]**	**IC_{50} (ng/mL)**	**Fold change[a]**	***p*-value[b]**	**IC_{50} (ng/mL)**	**Fold change[a]**	***p*-value[b]**
BA.1+S371F	7490.4	0.4	0.995	5130.3	0.9	0.568	602.8	0.6	0.89
BA.1+T376A	10,649.7	0.3	>0.999	4919.4	1.0	0.534	790.3	0.5	0.973
BA.1+D405N	>40,500	<0.1	>0.999	64.5	73.1	<0.001	133.7	2.9	0.003
BA.1+R408S	1092.8	2.9	0.001	1475.6	3.2	0.014	214.7	1.8	0.05
BA.1+G446	2890.8	1.1	0.396	9.0	524.8	<0.001	20.0	19.5	<0.001
BA.1+Q493	12.6	248.2	<0.001	16,393.1	0.3	0.998	20.1	19.3	<0.001
BA.1+G496	3112.4	1.0	0.49	134.8	35.0	<0.001	142.5	2.7	0.004
BA.1+G446+Q493	11.5	272.4	<0.001	34.5	136.8	<0.001	8.2	47.7	<0.001
BA.2+Q493	16.1	943.5	<0.001	2.3	0.8	0.696	4.4	0.8	0.749

[a]IC_{50} parental subvariant/IC_{50} mutant. [b]*p*-value of IC_{50} comparison of mutant versus parental subvariant.

IC_{50}, 50% inhibitory concentration; mAbs, monoclonal antibodies.

SUPPLEMENTAL TABLE 3 Plasmids encoding for the SARS-CoV-2 spike of Omicron subvariants

Subvariant	Mutations[a]
BA.1	A67V:HV69-:T95I:G142D:VYY143-:N211-:L212I:ins214EPE:G339D:S371L:S373P:S375F:K417N:N440K:G446S:S477N:T478K:E484A:Q493R:G496S:Q498R:N501Y:Y505H:T547K:D614G:H655Y:N679K:P681H:N764K:D796Y:N856K:Q954H:N969K:L981F
BA.1.1	A67V:HV69-:T95I:G142D:VYY143-:N211-:L212I:ins214EPE:G339D:R346K:S371L:S373P:S375F:K417N:N440K:G446S:S477N:T478K:E484A:Q493R:G496S:Q498R:N501Y:Y505H:T547K:D614G:H655Y:N679K:P681H:N764K:D796Y:N856K:Q954H:N969K:L981F
BA.2	T19I:LPP24-:A27S:G142D:V213G:G339D:S371F:S373P:S375F:T376A:D405N:R408S:K417N:N440K:S477N:T478K:E484A:Q493R:Q498R:N501Y:Y505H:D614G:H655Y:N679K:P681H:N764K:D796Y:Q954H:N969K
BA.2.12.1	T19I:LPP24-:A27S:G142D:V213G:G339D:S371F:S373P:S375F:T376A:D405N:R408S:K417N:N440K:L452Q:S477N:T478K:E484A:Q493R:Q498R:N501Y:Y505H:D614G:H655Y:N679K:P681H:S704L:N764K:D796Y:Q954H:N969K
BA.2.75	T19I:LPP24-:A27S:G142D:K147E:W152R:F157L:I210V:V213G:G257S:G339H:S371F:S373P:S375F:T376A:D405N:R408S:K417N:N440K:G446S:N460K:S477N:T478K:E484A:Q498R:N501Y:Y505H:D614G:H655Y:N679K:P681H:N764K:D796Y:Q954H:N969K
BA.4/BA.5	T19I:LPP24-:A27S:H69-:V70-:G142D:V213G:G339D:S371F:S373P:S375F:T376A:D405N:R408S:K417N:N440K:L452R:S477N:T478K:E484A:F486V:Q498R:N501Y:Y505H:D614G:H655Y:N679K:P681H:N764K:D796Y:Q954H:N969K

[a]Mutations relative to reference sequence Wuhan-Hu-1.

SUPPLEMENTAL TABLE 4 Plasmids encoding for SARS-CoV-2 spike mosaics incorporating Omicron subvariant-signatures in the background of BA.1 and BA.2

Mosaic	Mutations[a]
BA.1+S317F	A67V:HV69-:T95I:G142D:VYY143-:N211-:L212I:ins214EPE:G339D:S371F:S373P:S375F:K417N:N440K:G446S:S477N:T478K:E484A:Q493R:G496S:Q498R:N501Y:Y505H:T547K:D614G:H655Y:N679K:P681H:N764K:D796Y:N856K:Q954H:N969K:L981F
BA.1+T376A	A67V:HV69-:T95I:G142D:VYY143-:N211-:L212I:ins214EPE:G339D:S371L:S373P:S375F:T376A:K417N:N440K:G446S:S477N:T478K:E484A:Q493R:G496S:Q498R:N501Y:Y505H:T547K:D614G:H655Y:N679K:P681H:N764K:D796Y:N856K:Q954H:N969K:L981F
BA.1+D405N	A67V:HV69-:T95I:G142D:VYY143-:N211-:L212I:ins214EPE:G339D:S371L:S373P:S375F:D405N:K417N:N440K:G446S:S477N:T478K:E484A:Q493R:G496S:Q498R:N501Y:Y505H:T547K:D614G:H655Y:N679K:P681H:N764K:D796Y:N856K:Q954H:N969K:L981F
BA.1+R408S	A67V:HV69-:T95I:G142D:VYY143-:N211-:L212I:ins214EPE:G339D:S371L:S373P:S375F:R408S:K417N:N440K:G446S:S477N:T478K:E484A:Q493R:G496S:Q498R:N501Y:Y505H:T547K:D614G:H655Y:N679K:P681H:N764K:D796Y:N856K:Q954H:N969K:L981F
BA.1+G446	A67V:HV69-:T95I:G142D:VYY143-:N211-:L212I:ins214EPE:G339D:S371L:S373P:S375F:K417N:N440K:S477N:T478K:E484A:Q493R:G496S:Q498R:N501Y:Y505H:T547K:D614G:H655Y:N679K:P681H:N764K:D796Y:N856K:Q954H:N969K:L981F
BA.1+Q493	A67V:HV69-:T95I:G142D:VYY143-:N211-:L212I:ins214EPE:G339D:S371L:S373P:S375F:K417N:N440K:G446S:S477N:T478K:E484A:G496S:Q498R:N501Y:Y505H:T547K:D614G:H655Y:N679K:P681H:N764K:D796Y:N856K:Q954H:N969K:L981F
BA.1+G496	A67V:HV69-:T95I:G142D:VYY143-:N211-:L212I:ins214EPE:G339D:S371L:S373P:S375F:K417N:N440K:G446S:S477N:T478K:E484A:Q493R:Q498R:N501Y:Y505H:T547K:D614G:H655Y:N679K:P681H:N764K:D796Y:N856K:Q954H:N969K:L981F
BA.1+G446+Q493	A67V:HV69-:T95I:G142D:VYY143-:N211-:L212I:ins214EPE:G339D:S371L:S373P:S375F:K417N:N440K:S477N:T478K:E484A:G496S:Q498R:N501Y:Y505H:T547K:D614G:H655Y:N679K:P681H:N764K:D796Y:N856K:Q954H:N969K:L981F
BA.2+Q493	T19I:LPP24-:A27S:G142D:V213G:G339D:S371F:S373P:S375F:T376A:D405N:R408S:K417N:N440K:S477N:T478K:E484A:Q498R:N501Y:Y505H:D614G:H655Y:N679K:P681H:N764K:D796Y:Q954H:N969K

[a]Mutations relative to reference sequence Wuhan-Hu-1.

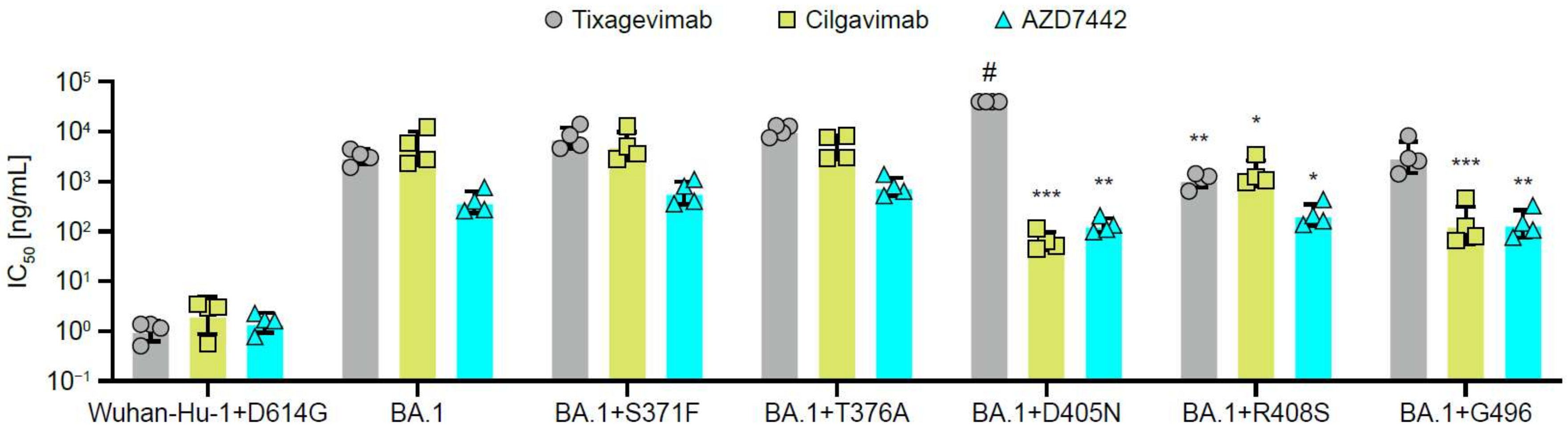

SUPPLEMENTAL FIG. 1 BA.1 mosaic mutants with lower impact on susceptibility to AZD7442. 50% inhibitory concentration (IC_{50}) values of mosaic mutants obtained in the microneutralization assay using SARS-CoV-2 spike pseudotyped lentiviral particles. See Fig. 4a for sequence of mosaic mutants. #: IC_{50} values greater than upper limit of quantitation. *: *p*-value 0.05–0.01; **: *p*-value 0.01–0.001; *** *p*-value <0.001.

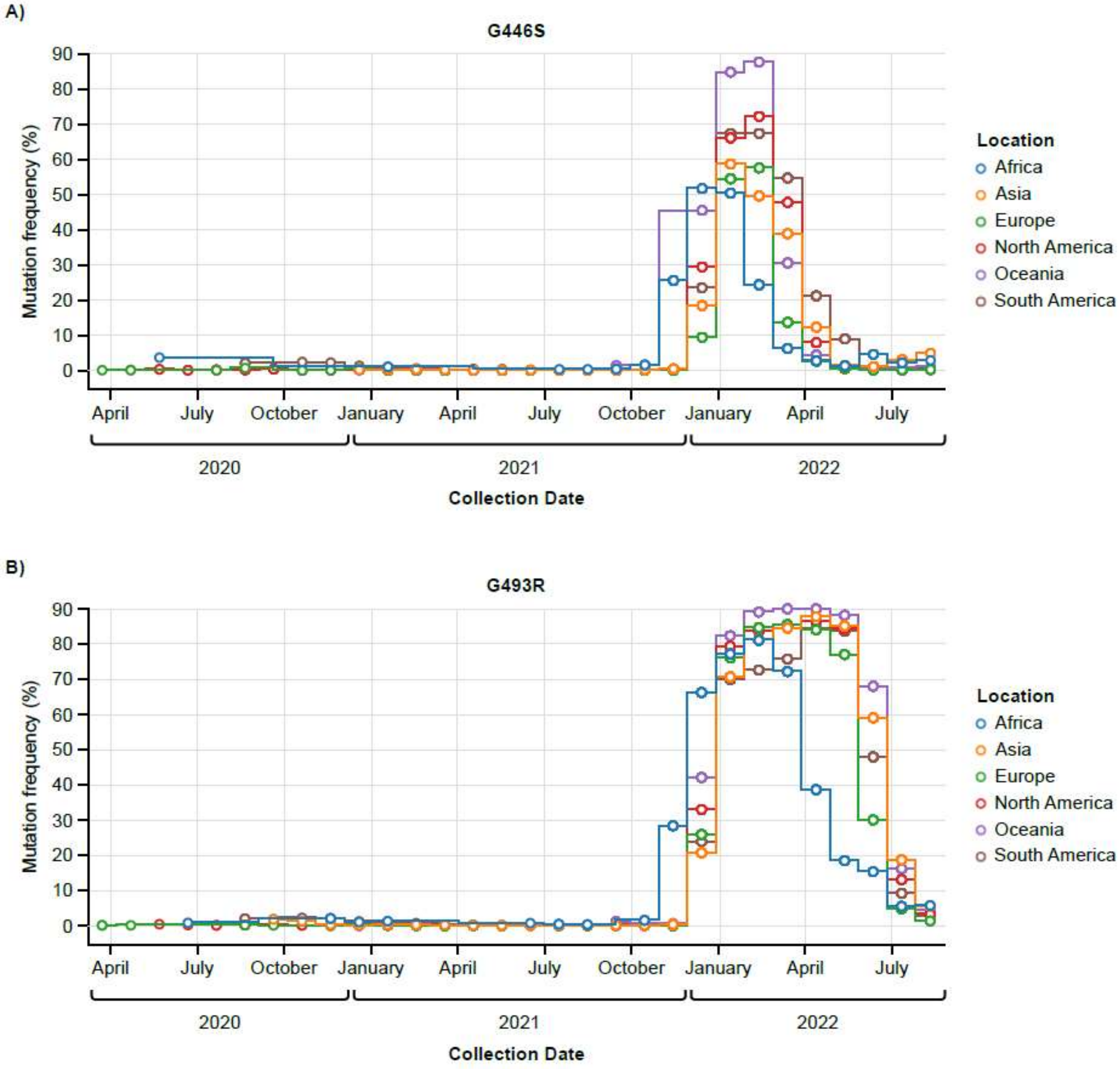

SUPPLEMENTAL FIG. 2 Global dynamics of SARS-CoV-2 spike mutations G446S and Q493R. Y-axis depicts the monthly regional frequency of mutations (A) G446S and (B) Q493R based on date of sequence collection (x-axis) from the beginning of the SARS-CoV-2 pandemic to July 2022. Analysis conducted in the COVIDCG pipeline (1) based on sequence data deposited in GISAID database (2). See Materials and Methods for details.

References

1. Chen AT, Altschuler K, Zhan SH, Chan YA, Deverman BE. 2021. COVID-19 CG enables SARS-CoV-2 mutation and lineage tracking by locations and dates of interest. Elife 10:e63409.
2. Shu Y, McCauley J. 2017. GISAID: Global initiative on sharing all influenza data - from vision to reality. Euro Surveill 22:30494.
